# Monthly Botanical Report

**Published:** 1811-02

**Authors:** 


### MONTHLY BOTANICAL REPORT.

The Botanical Magazine for last month contains,

Aloë *radula* of Jacquin, the *attenuata* of Haworth. Of the smaller aloës, which are better suited to the size of the work, Mr. Edwards has given some excellent specimens of his superior skill as an artist. Indeed we have seldom witnessed any thing superior, even in the splendid botanical productions of the Paris Press.

Aloë *saponaria* (*β. latifolia*;) one of the old varieties of *perfoliata*; a very large species, but of which a good idea is given by the insertion of a diminished outline, representingthe habit of the whole plant. Mr. Ker has in this, as in all the tribe, taken great pains to elucidate the confused synonimy. He informs us that the smaller variety of this (*β minor*) is the *umbellata* of Decandolle, excluding all his synonyms, which belong to A. *picta*; and is also the *picta* (*minor*) of the new edition of the Hortus Kewensis, as far as regards the synonym of the late edition, but that those quoted from Linnæus næus and Dillenius belong to A. *picta*. Although this species has been called American aloe, and Carolina aloe, it is not a native of America, but of the Cape.

Tamus *eliphantipes*. A female plant of the Cape Bryony, from Mr. Knight's collection, in the King's-road, Chelsea. A male plant flowered in 1783 in the Kew garden, from which M. L'Heritier had a drawing taken for his sertum anglicum; the engraving, however, though quoted in books, was never published; nor is there any figure of it, that we know of, extant. To those who have never seen this very singular plant, Mr. Edwards's outline sketch behind the flowering stem, will not be easily understood. It represents the curious root stock, which rises above the surface of the ground, and somewhat resembles a hemispherical section of the trunk, or rather of a warty excrescence of some old tree. In this apparently lifeless state it sometimes remains many months, now and then putting forth climbing stems, bearing alternate cordate leaves, with here and there bunches of flowers in the axils of their footstalks, much in the same manner as the common black bryony. It is this shapeless, massive rootstalk that has occasioned its being called, by the inhabitants of the Cape, the Elephants-foot.

Hermannia *tenuifolia*; a new species, which is probably lost to our gardens, as the drawing was taken in Mr. Curtis's time, and except an imperfect specimen in the Banksian herbarium, Dr. Sims has not been able to find any thing respecting its existence.

Hermannia *flammea*; a beautiful little shrub from Mr. Knight's collection. Although we are presented with a good drawing of this plant, with flowers fully expanded, yet it is rarely seen in this state, we could have wished that some of the flowers at least had been represented in their very remarkably light-twisted state, in which they look almost as if the tips had been rounded off with a pair of scissars. Mr. Andrews, in the Botanist's Repository, though his figure is otherwise very indifferent, has seized this peculiarity. The nocturnal fragrance of the flowers adds to the value of this plant.

Astragalus *sinicus*. It seems remarkable that this pretty little annual should never have been before figured, though it has been at times in our gardens for forty years past.

Tropæolum *peregrinum*. This is at present a scarce and considered as a tender plant, but being a native of the same country as the common Tropæolum *majus*, there seems no reason why it should not become as hardy as that, which is now almost naturalized to our clime; for Miller says, that this last will sow itself and come up spontaneously the following summer, in favourable situations.

There are several species of this singular genus recorded in the Flora Peruviana, and it is not improbable that distinct plants have been confounded under the name of T. *peregrinum*; Dr. Sims doubts, if Feuillée's plant, cited as a synonym of this by all preceding writers, be the same. However this may be, there is no doubt respecting Jacquin's plant, well figured in his Hortus Schoenbrunensis.

The

The English Flora for last month offers to our notice:—

Rubus *saxatilis*, as a native of high mountains in the northern part of the island. The specimen from which the drawing was taken, was gathered by Mr. Borret, at Roslin, famous for its antique chapel, and delicious strawberries.

Brassica *campestris*. Dr. Smith remarks, that great uncertainty has existed among British authors, even from the time of Ray, respecting this plant.

Hudson's *campestris* is a mere yellow variety of orientalis. According to Mr. Edw. Forster, this is the common Wild Navew, growing abundantly by the sides of rivers, marsh ditches, &c. and that the B. *Napus* of English Flora, is the rape or cole-seed, so commonly cultivated.

Hieracium *prenanthoides*. Brought from Scotland many years ago, by Mr. Dixon, and has been ever since in Mr. E. Forster's garden, from whence this drawing was made. Dr. Smith observes, that in the Flora Britannica he had confounded this plant with *denticulatum*. There continues to be great difficulty in settling the species of this genus. Dr. Smith believes, from the dried specimens he has received from Mr. G. Don, that the Scottish species are not yet all determined, but that the greatest attention to living plants can alone enable him to reduce them to order. When that is accomplished, he possesses ample materials for settling their synonyms

Carex *Micheliana*, introduced into Dr. Smith's Flora Britannica, he is now convinced is only a variety of *recurva*, as which he first received it from Dr. Beattie of Aberdeen. The name of *Micheliana* being therefore superfluous, as Dr. Smith himself allows, ought not surely to have been suffered to stand as the title of this mere variety of *recurva*.

